# Correction: Dexrazoxane does not mitigate early vascular toxicity induced by doxorubicin in mice

**DOI:** 10.1371/journal.pone.0296372

**Published:** 2023-12-20

**Authors:** The PLOS ONE Staff

## Notice of Republication

An incorrect version of S2 Figure was published and the publisher apologizes for this error. This article was republished on December 11, 2023 to correct this error. Please download this article again to view the correct version.Reference
