# Supplementary figures and images for: Dexrazoxane does not mitigate early vascular toxicity induced by doxorubicin in mice
Source: PLoS One. 2023 Nov 28;18(11):e0294848. doi: 10.1371/journal.pone.0294848 (PMC10684076; doi:10.1371/journal.pone.0294848)

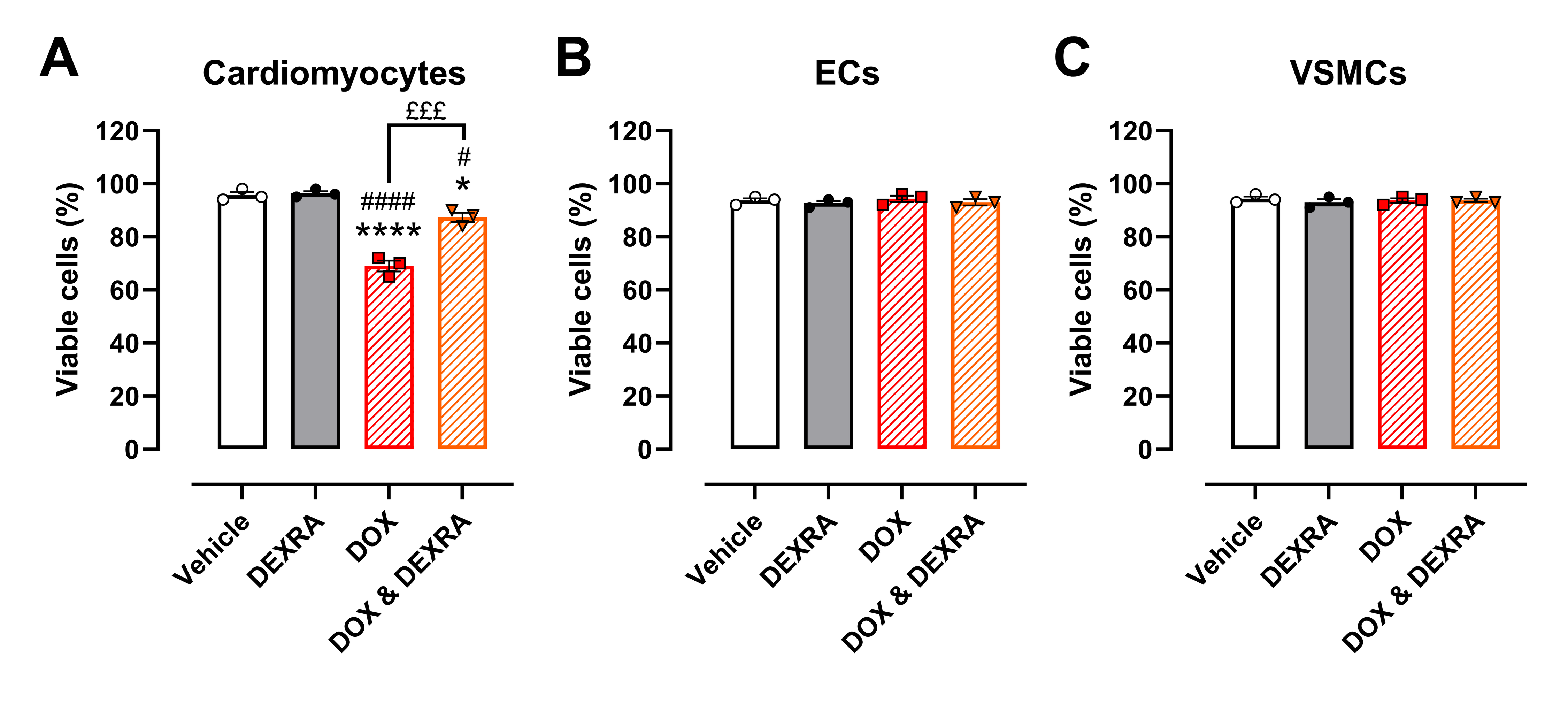

Supplement: S1 Fig — DOX-treated cardiomyocytes showed lower viability compared to the vehicle- and DEXRA-treated groups, but this was attenuated in the presence of DEXRA (A). DOX did not affect viability in ECs (B) and VSMCs (C). For all panels: One-way ANOVA with Tukey’s multiple comparisons test; n = 3 in each group. For A: *, ****p<0.05, 0.0001 compared to vehicle; #, #### p<0.05, 0.0001 compared to DEXRA group; £££ p<0.001 between DOX and DOX with DEXRA groups. For B & C: p>0.05 for all groups. (TIF) [file pone.0294848.s001.tif]

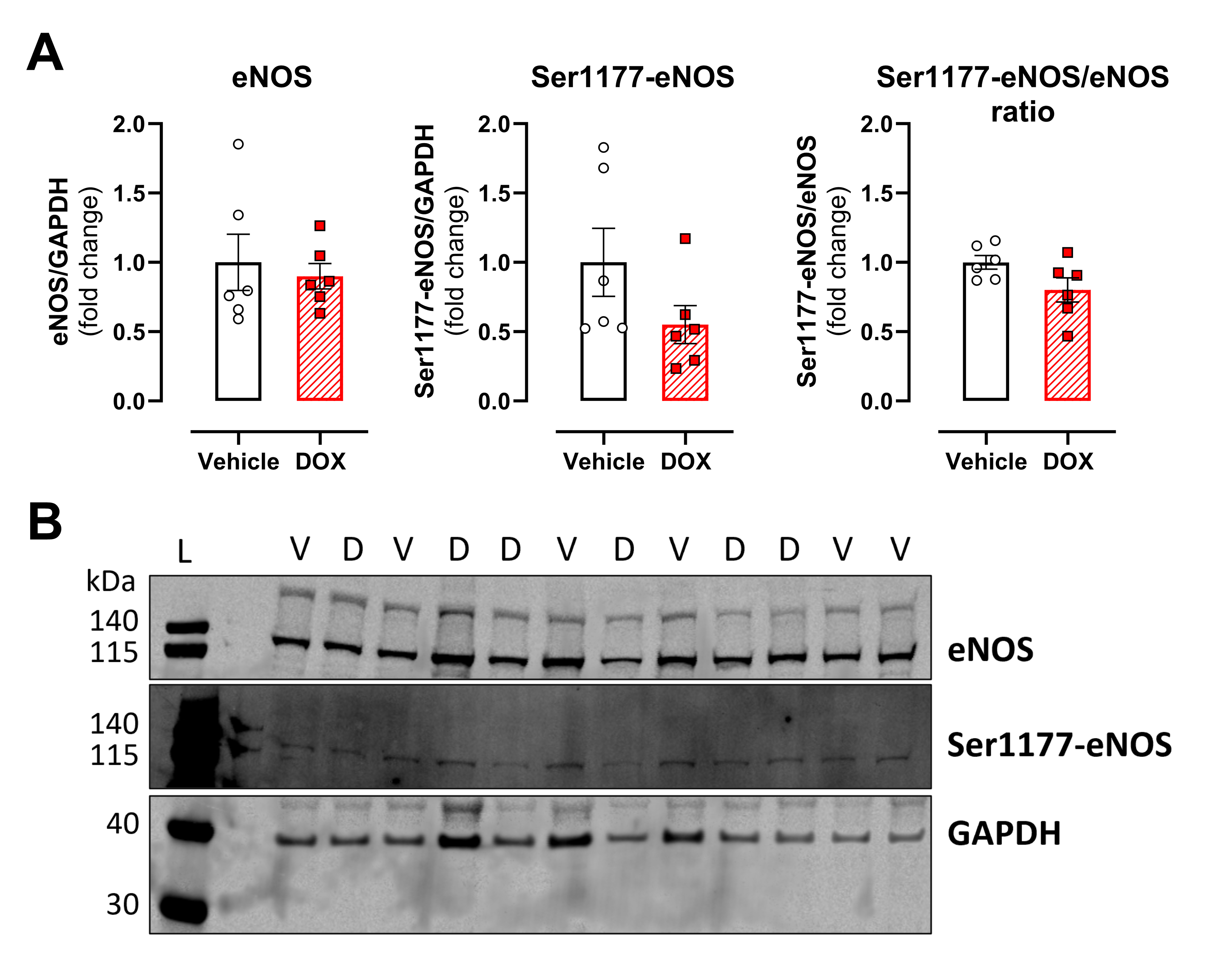

Supplement: S2 Fig — DOX does not alter eNOS nor Ser1177-eNOS levels shortly after administration (A). Representative blot for panel A (B). For A: Mann-Whitney U test. n = 6 in each group. p>0.05 for vehicle vs. DOX groups. For B: “L”, “V” and “D” stand for ladder, vehicle and DOX, respectively. (TIF) [file pone.0294848.s002.tif]
