# Supplementary material for: Dexrazoxane does not mitigate early vascular toxicity induced by doxorubicin in mice
Source: PLoS One. 2023 Nov 28;18(11):e0294848. doi: 10.1371/journal.pone.0294848 (PMC10684076; doi:10.1371/journal.pone.0294848)

Blot 1 – GAPDH

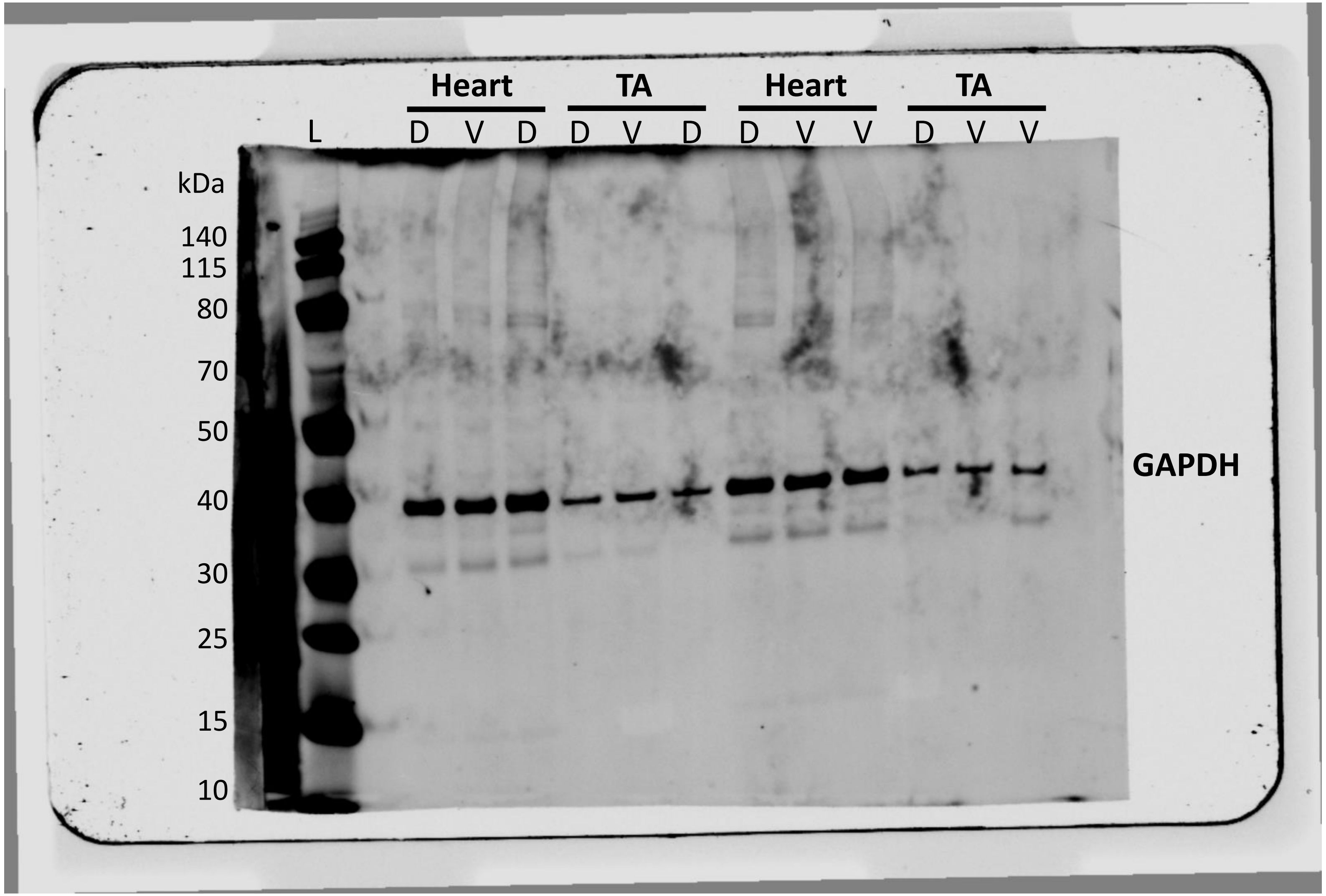

L, D and V stand for ladder, DOX and vehicle, respectively

Blot 1 – TOP-IIβ & GAPDH

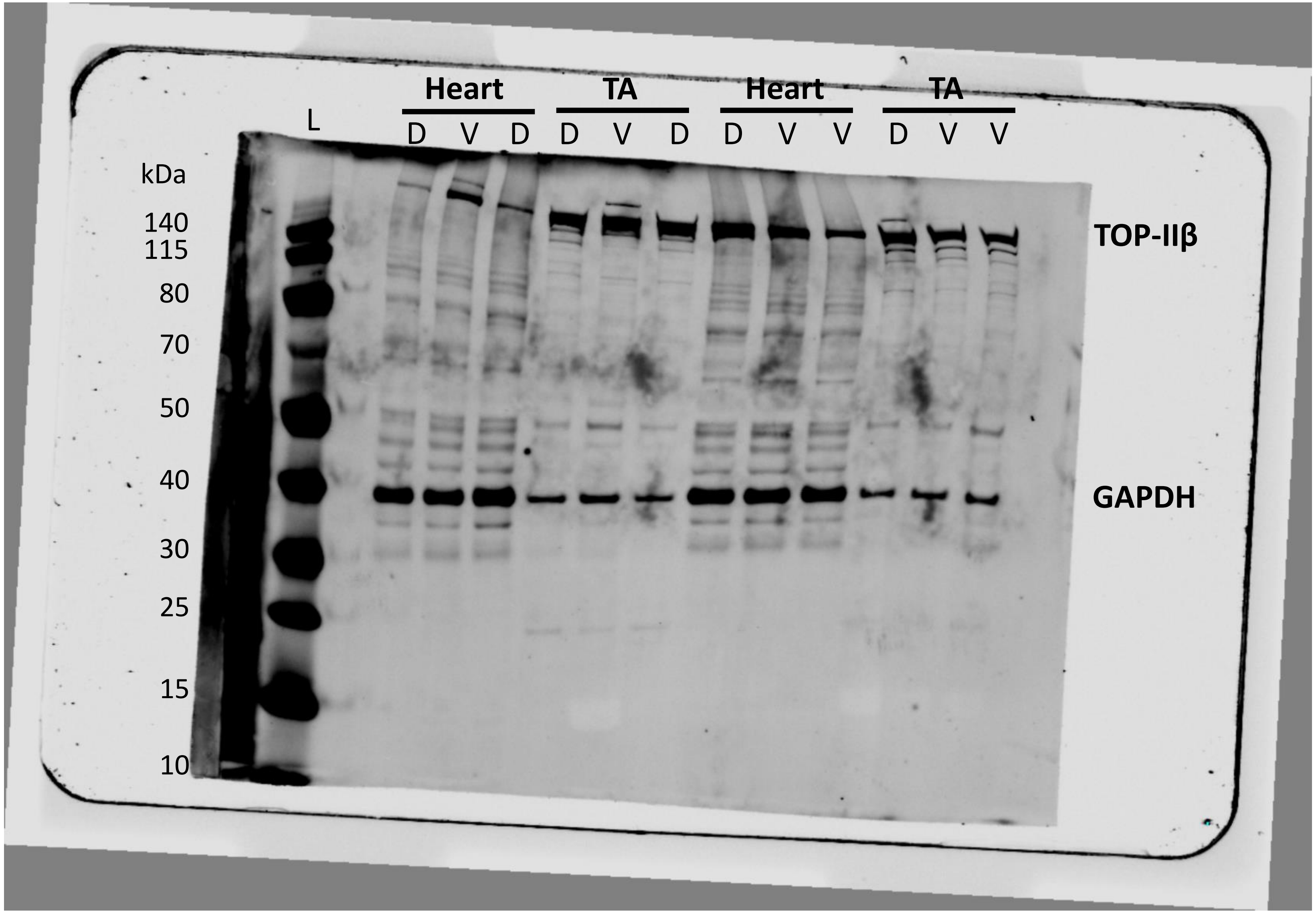

L, D and V stand for ladder, DOX and vehicle, respectively

Blot 2 – GAPDH

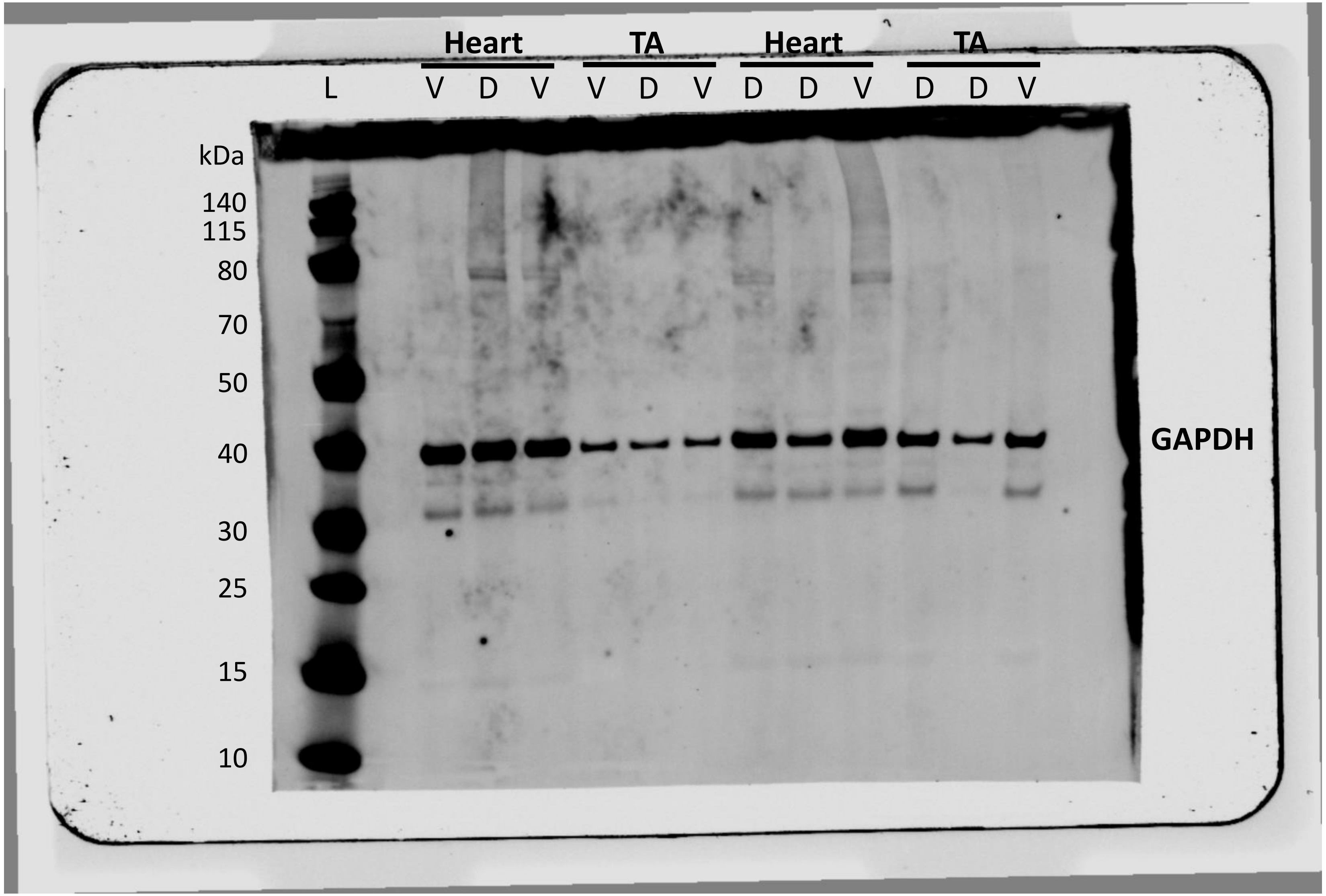

L, D and V stand for ladder, DOX and vehicle, respectively

Blot 2 – TOP-IIβ & GAPDH

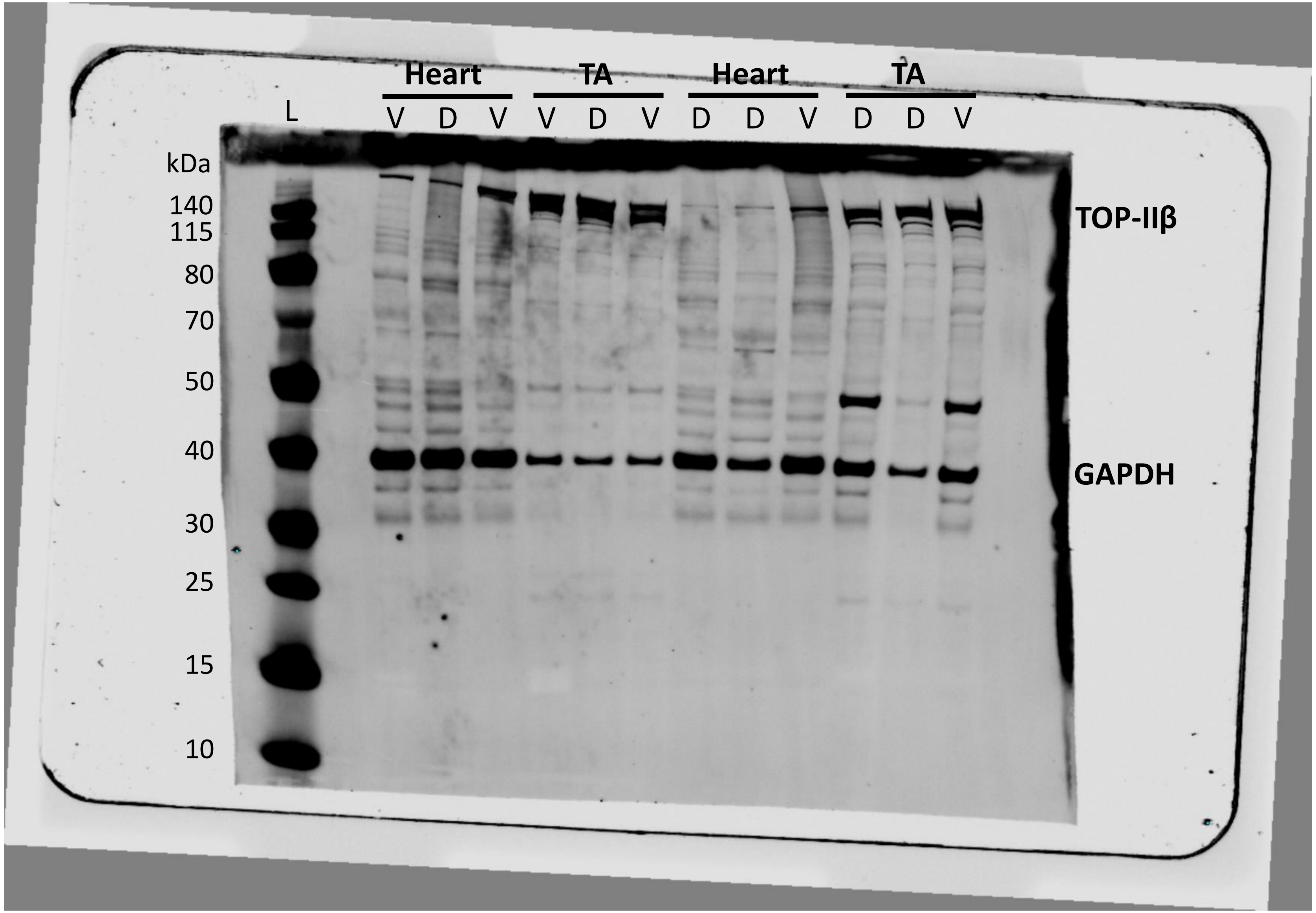

L, D and V stand for ladder, DOX and vehicle, respectively

Blot 3 – eNOS & GAPDH

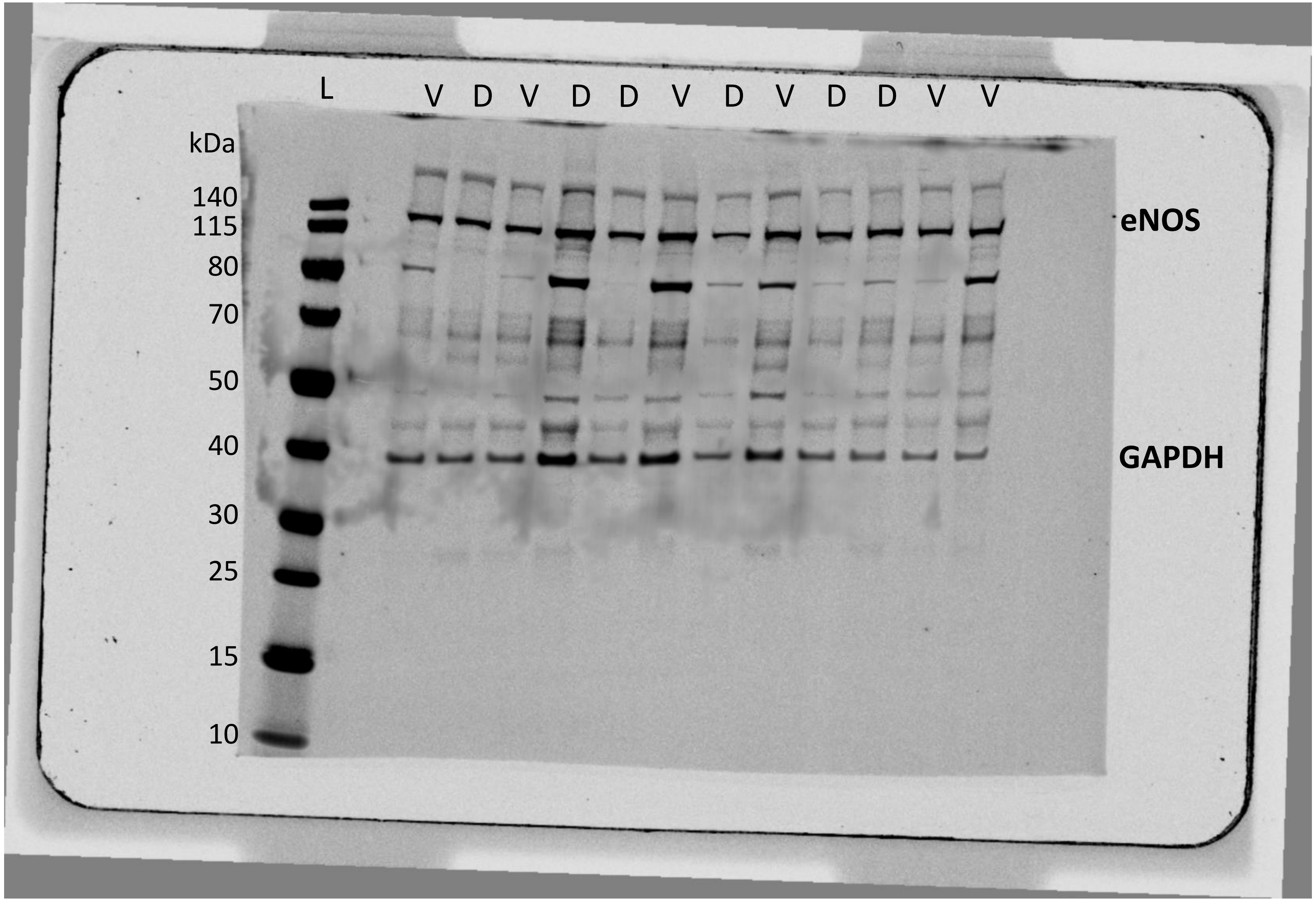

L, D and V stand for ladder, DOX and vehicle, respectively

Blot 3 – Ser1177-eNOS & GAPDH

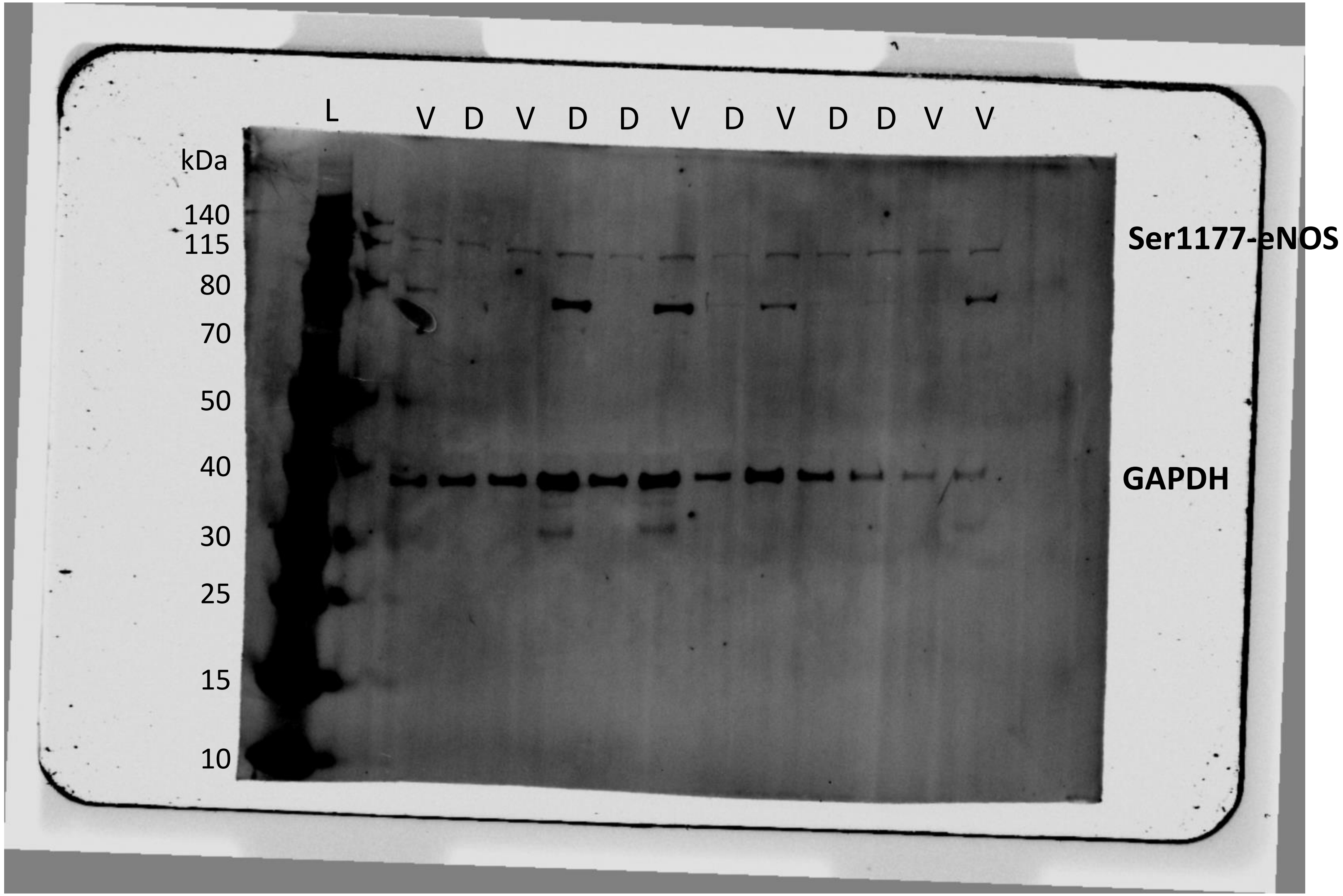

L, D and V stand for ladder, DOX and vehicle, respectively

Shown in Figure 4

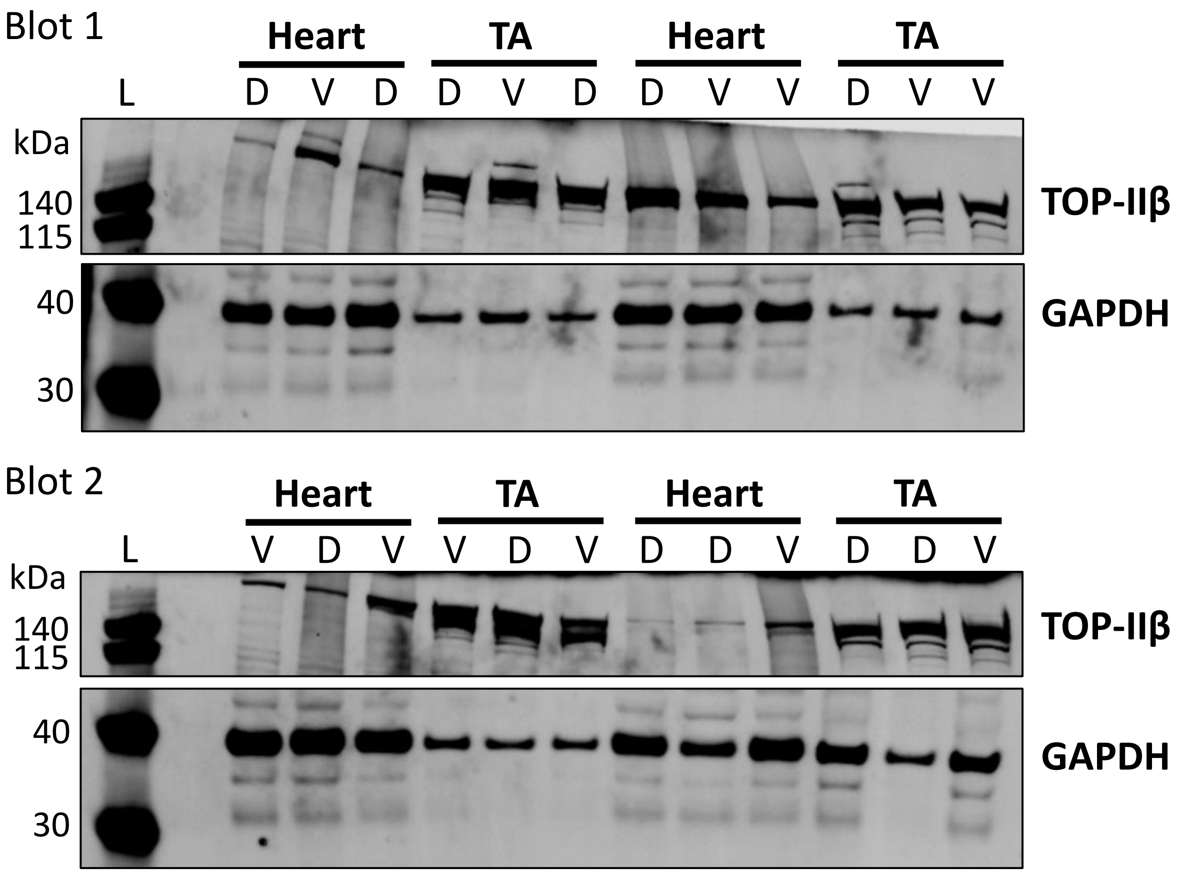

Shown in Supplementary Figure 2

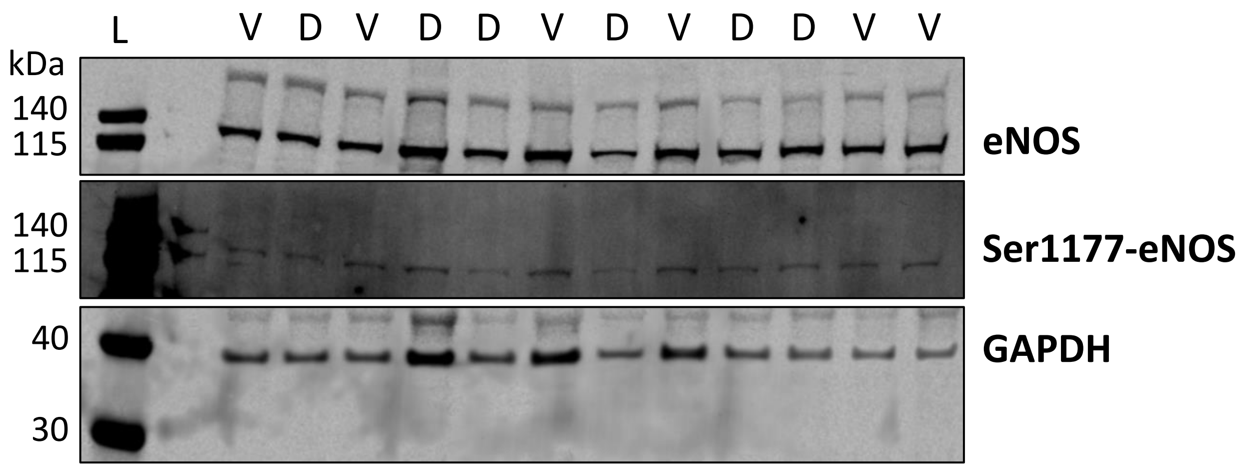

Supplement: S1 Raw images — (PDF) [file pone.0294848.s004.pdf]
